# Topological length of white matter connections predicts their rate of atrophy in premanifest Huntington’s disease

**DOI:** 10.1172/jci.insight.92641

**Published:** 2017-04-20

**Authors:** Peter McColgan, Kiran K. Seunarine, Sarah Gregory, Adeel Razi, Marina Papoutsi, Jeffrey D. Long, James A. Mills, Eileanoir Johnson, Alexandra Durr, Raymund A.C. Roos, Blair R. Leavitt, Julie C. Stout, Rachael I. Scahill, Chris A. Clark, Geraint Rees, Sarah J. Tabrizi, the Track-On HD Investigators

**Affiliations:** 1 Huntington’s Disease Centre, Department of Neurodegenerative Disease,; 2 Developmental Imaging and Biophysics Section, UCL Institute of Child Health, London, United Kingdom.; 3 Wellcome Trust Centre for Neuroimaging, UCL Institute of Neurology, London, United Kingdom.; 4 Department of Electronic Engineering, NED University of Engineering and Technology, Karachi, Pakistan.; 5 Department of Psychiatry,; 6 Department of Biostatistics, University of Iowa, Iowa City, Iowa, USA.; 7 APHP Department of Genetics, University Hospital Pitié-Salpêtrière, and ICM (Brain and Spine Institute) INSERM U1127, CNRS UMR7225, Sorbonne Universités – UPMC Paris VI UMR_S1127, Paris, France.; 8 Department of Neurology, Leiden University Medical Centre, Leiden, Netherlands.; 9 Centre for Molecular Medicine and Therapeutics, Department of Medical Genetics, University of British Columbia, Vancouver British Columbia, Canada.; 10 School of Psychological Sciences, Monash University, Australia.; 11 National Hospital for Neurology and Neurosurgery, Queen Square, London, United Kingdom.; 12 The Track-On HD Investigators are detailed in the Supplemental Acknowledgments.

## Abstract

We lack a mechanistic explanation for the stereotyped pattern of white matter loss seen in Huntington’s disease (HD). While the earliest white matter changes are seen around the striatum, within the corpus callosum, and in the posterior white matter tracts, the order in which these changes occur and why these white matter connections are specifically vulnerable is unclear. Here, we use diffusion tractography in a longitudinal cohort of individuals yet to develop clinical symptoms of HD to identify a hierarchy of vulnerability, where the topological length of white matter connections between a brain area and its neighbors predicts the rate of atrophy over 24 months. This demonstrates a new principle underlying neurodegeneration in HD, whereby brain connections with the greatest topological length are the first to suffer damage that can account for the stereotyped pattern of white matter loss observed in premanifest HD.

## Abstract 2

Diffusion tractography in a longitudinal cohort demonstrates that topological length of white matter connections can account for white matter loss patterns in premanifest Huntington’s disease.

## Introduction

Huntington’s disease (HD) is a fully penetrant neurodegenerative disease caused by a dominantly inherited CAG trinucleotide repeat expansion in the Huntingtin gene (*HTT*). This results in the production of a mutant huntingtin protein with an abnormally long polyglutamine repeat (1). The full penetrance of HD makes it possible to predict who will develop the disease many years before symptom onset (2). In the premanifest stage, prior to disease onset, individuals show gray matter loss in the striatum and white matter volume loss around the striatum, within the corpus callosum and in the posterior white matter tracts (3). Micro-structural white matter changes have also been demonstrated in these regions in premanifest HD (preHD) (4–6).

While these findings provide strong evidence for the earliest white matter changes in preHD, it is still unclear the order in which white matter connections degenerate and why some white matter connections are more vulnerable than others. With the antisense oligonucleotide (ASO) huntingtin lowering trial (7) currently underway, there is an urgent need to understand the time course of white matter changes and the mechanisms that drive them so that brain areas can be identified that may benefit from the highest concentrations of ASO.

Medium spiny neurons (MSNs) of the striatum are selectively vulnerable to the effects of mutant huntingtin (8). One theory for this selective vulnerability is that the high-energy demands of MSNs (9) makes them particularly susceptible to mitochondrial dysfunction induced by the presence of mutant huntingtin (10). Given that long-range white matter connections are the most metabolically active (11) and mutant huntingtin causes metabolic disturbance through mitochondrial dysfunction (12), we hypothesized that the topological length of white matter connections would determine their vulnerability in preHD.

To test this hypothesis, we studied white matter connectivity in a longitudinal cohort of preHD participants over 24 months. We classified white matter connections into subtypes based on connections between the cortex and striatum (cortico-striatal), between cerebral hemispheres (interhemispheric), within cerebral hemispheres (intrahemispheric), and within cortical modules (intramodular). We examined how these connections differ from controls both cross-sectionally and longitudinally. To test whether the topological length of white matter connections determined vulnerability, we then investigated whether shortest weighted path length, a network measure of distance between a pair of brain regions, determined cross-sectional and/or longitudinal change.

## Results

### White matter connection subtypes.

**Figure 1:**
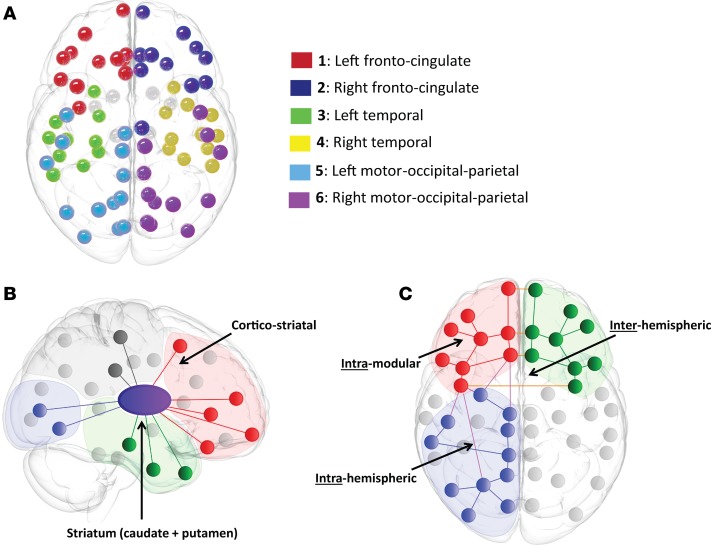
Module assignment and connection types. (**A**) Module assignment derived using the Louvain community detection algorithm on the average control baseline network. This results in 6 putative cortical modules: fronto-cingulate, temporal, and motor-occipital-parietal. (**B**) Cortico-striatal connections. For the connectome analysis, these are defined as the sum of streamline weights (connection strength) from the caudate and putamen to a cortical module. (**C**) Connection types in the cortex. Intramodular: sum of streamline weights (connection strength) within the same module (Red-Red). Intrahemispheric: sum of streamline weights (connection strength) between modules in the same hemisphere (Blue-Red). Interhemispheric: sum of streamline weights (connection strength) between modules in different hemispheres (Red-Green).

Using a data-driven community Louvain approach (13), cortical regions of interest (ROIs) were assigned to a cortical module, where each module represents a set of cortical ROIs that have maximum connections with each other but minimum connections with all other regions outside the module. This resulted in 6 modules, 3 in the left hemisphere and 3 in the right hemisphere. Module assignment was as follows: module 1, left-sided frontal regions, left caudal anterior and rostral anterior cingulate regions, and left insula; module 2, right-sided frontal regions and right caudal anterior, rostral anterior, and posterior cingulate regions; module 3, left temporal regions; module 4, right temporal regions and right insula; module 5, left motor, occipital, parietal, and isthmus and posterior cingulate regions; and module 6, right motor, occipital, and parietal and left isthmus cingulate regions (Figure 1A and Supplemental Table 1; supplemental material available online with this article; https://doi.org/10.1172/jci.insight.92641DS1 for details). To ensure that our results did not depend on module partitioning, we repeated the connection-length atrophy analysis with 4 and 8 module partitions. Key results were robust to varying the number of module partitions (see Methods and Supplemental Figure 1 and Supplemental Figure 2).

Connections were then classified into subtypes: these included 6 cortico-striatal connections (between the striatum [caudate and putamen]and each cortical module), 9 interhemispheric connections (between modules in different hemispheres), 6 intrahemispheric connections (between modules in the same hemisphere), and 6 intramodular connections (within each module). The strength of each connection is represented by the sum of its connection weights. See Figure 1, B and C, for an illustration of connection subtypes.

### Hierarchy of white matter connection vulnerability in preHD vs.

*controls*. In order to maximize the robustness of our results, the cortico-striatal connections were analyzed using 2 complimentary tractography approaches: voxel connectivity profiles (VCPs) (14) and a connectome (15) approach. For the VCPs, streamlines are seeded in the striatum (caudate and putamen) and project to multiple regions in the cortex. Cortico-striatal connections are then normalized by the volumes of the ROIs they connect, thus taking into account gray matter atrophy both in the cortex and striatum. In the connectome approach, streamlines are seeded throughout the white matter and terminate when they reach gray matter regions, such that connections between all pairs of ROIs are investigated and are independent of the origin of specific tracts.

Linear mixed effects regression (LMER) was used to investigate longitudinal group differences in connection strength between preHD and controls (differences in slopes), with baseline (study entry) cross-sectional differences being represented by intercept differences. Baseline covariates included age at study entry, sex, education, and study site. The time metric was “time on study (years): 0 = baseline. Z-ratios of fixed effects estimates were used to test the null hypothesis of no group differences. The FDR correction was used for multiple comparisons, and unadjusted *P* values or FDR-adjusted *q* values are reported depending on the effects of interest (*P* < 0.05 and *q* < 0.05).

The first question we asked was how different connection subtypes differ between groups at baseline. This was addressed by testing for intercept differences between the groups (preHD minus controls). For cortico-striatal connections, both VCP and connectome analyses showed statistically significant (*q* < 0.05) weaker connection strength in preHD relative to controls for all 6 cortico-striatal connections (100%). See Table 1 for connectome results and Supplemental Table 2 for VCP results.

For interhemispheric connections, preHD showed significantly (*q* < 0.05) weaker connection strength compared with controls in 6 connections (67%). These included the connections between the posterior motor-occipital parietal modules and their connections with the anterior fronto-cingulate modules. Connections between left and right temporal modules and left and right fronto-cingulate modules were also affected.

**Figure 2:**
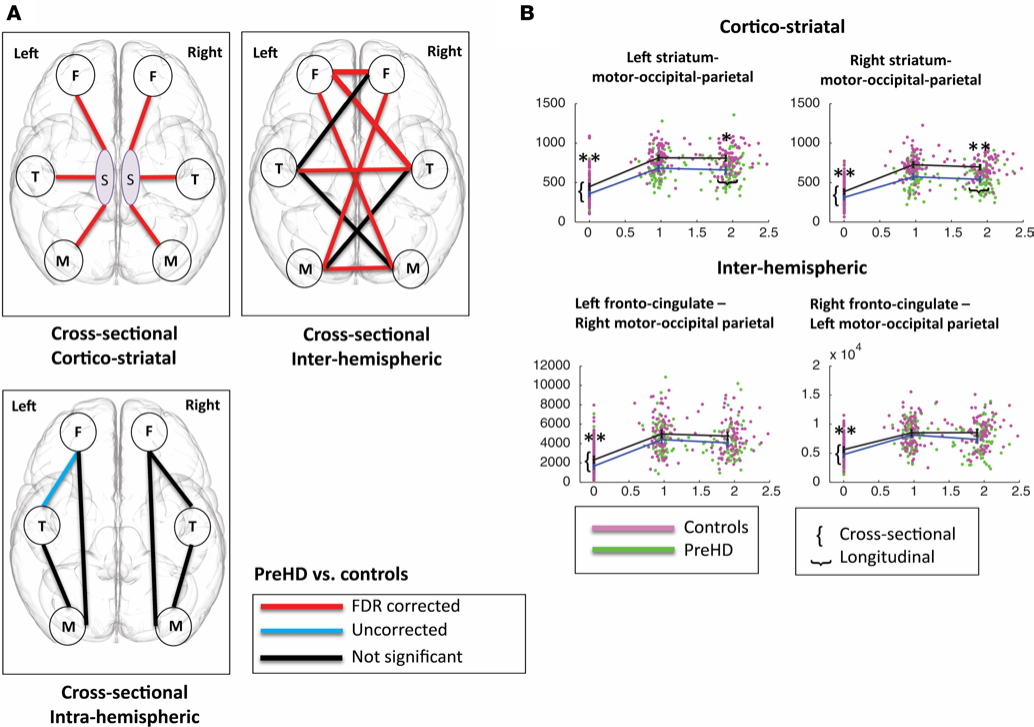
Hierarchy of connection vulnerability. Mixed linear model results for connectome analysis: preHD vs. controls. Cortico-striatal connections are most affected, followed by interhemispheric connections and then intrahemispheric connections. (**A**) Cross-sectional cortico-striatal figure illustrates cross-sectional cortico-striatal differences where premanifest Huntington’s disease (preHD) show reduced connection strength between the striatum (caudate and putamen) and cortical modules. Cross-sectional interhemispheric figure illustrates cross-sectional interhemispheric differences, where preHD show reduced connection strength between left and right hemisphere cortical modules. Cross-sectional intrahemispheric figure illustrates cross-sectional intrahemispheric differences, where preHD show reduced connection strength between left cortical modules and right cortical modules separately (F, front-cingulate; T, temporal; M, motor-occipital-parietal; S, striatum). (**B**) Connection strength at baseline and 24-month followup for cortico-striatal and interhemispheric connections. Cross-sectional group difference was defined as the intercept main effect of group in the full linear mixed effects model. Longitudinal change was defined as a significantly superior fit for the full Linear mixed effects regression (LMER) compared with the reduced LMER omitting group by time interaction (see Methods for further details), which means that the group by time interaction effect was significant and preHD patients show more connectivity loss over time compared with controls. Data presented as dot plots with group means ± 95% CI at each time point for control (magenta), preHD (green). *P < 0.05, **P <0.01. *y*-axis: connection strength, *x*-axis: follow-up time in years. *y*-axis differs between graphs, as connection strengths vary in range between different connections (486 data points displayed for each figure).

No significant cross-sectional FDR-corrected group differences were seen in intrahemispheric (0%) or intramodular connections (0%). PreHD showed weaker connection strength (*P* < 0.05) for one intrahemispheric connection and one intramodular connection compared with controls. See Figure 2 and Table 1 for the cross-sectional results.

In summary, the weakest preHD connection strength at baseline was for cortico-striatal connections, followed by interhemispheric connections, intrahemispheric, and intramodular connections. This suggests a temporal hierarchical pattern of degeneration. To verify whether this was the case, we looked at the group rate of change over time (group slopes) and whether the same connections showed greater change over time.

### Rate of change in connection strength over time in preHD vs.

*controls*. For cortico-striatal connections, both VCPs and connectome analyses showed significant decreases in connection strength over time in preHD relative to controls for the bilateral striatal motor-occipital-parietal connections, although for the left connection in the connectome analysis and the right connection in the VCP analysis, this reduction was only seen for *P* < 0.05. See Figure 2B and Table 2 for connectome results. The VCP analysis also showed significant reductions in the striatum fronto-cingulate connections bilaterally, although only for *P* < 0.05 in the left, while the connectome analysis revealed (*P* < 0.05) a decrease in connection strength over time in the left striatum-temporal connection in the preHD group compared with controls (Table 2).

No significant longitudinal changes were seen for interhemispheric connections (Supplemental Table 3). We did find (only for *P* < 0.05) a longitudinal increase in connection strength in the right fronto-cingulate to right temporal intrahemispheric connection in preHD relative to controls (Table 2). No significant longitudinal changes were seen for intramodular connections (see Supplemental Table 3).

In summary, over 24 months, only cortico-striatal connections significantly degenerated in the preHD group relative to controls. We hypothesized that the reason these connections might be so vulnerable is because of their length. Therefore, in the next stage of our study, we performed an analysis to test the relationship between connection length and connection atrophy.

### Relationship between connection length and connection subtype in healthy controls.

**Figure 3:**
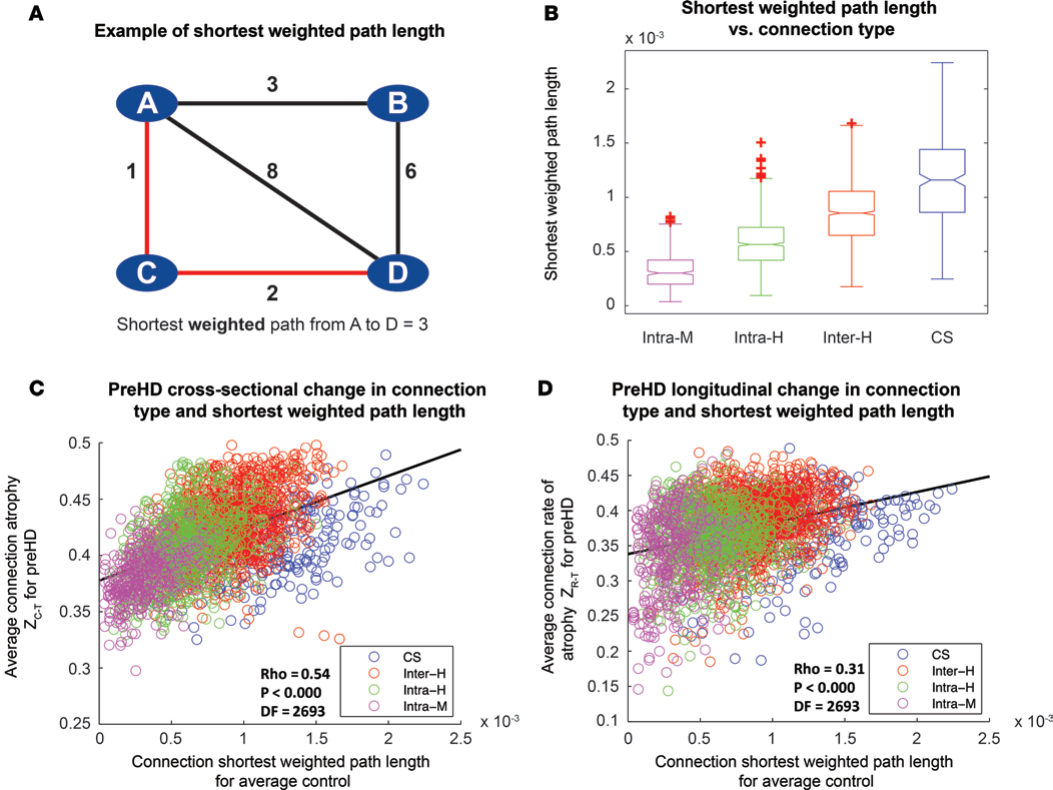
Connection length varies according to connection type and correlates with rate of connection degeneration over 2 years in premanifest Huntington’s Disease (preHD). (**A**) Illustration of shortest weighted path length between A and D in an example network. Numbers represent connection weights. When calculating shortest weighted path, length connections are weighted by the inverse of streamline weights, as stronger connections represent shorter paths in graph theory. (**B**) Comparison of shortest weighted path length for different classes of connection. Intra-M, intramodular (magenta); Intra-H, intrahemispheric (green); Inter-H, interhemispheric (red); CS, cortico-striatal (blue). Lower line, minimum; upper line, maximum; middle-box line, median; lower-box line, 1^st^ quartile; upper-box line: 3^rd^ quartile. Red crosses indicate outliers. (**C**) Cross-sectional analysis: Z-scores, denoting loss of connection strength, were transformed into positive atrophy measures using a logistic transform. Average transformed connection strength Z-score for preHD participants was plotted against connection-weighted path length for average control, and Spearman rank correlations were performed. Connections are color coded according to type**.** (**D**) Longitudinal analysis: Z-scores, denoting connection rate of atrophy over 3 time points, were transformed into a positive rate of atrophy measure using a logistic transform. Average transformed connection rate of change Z-scores for preHD participants were plotted against connection-weighted path length for average control, and Spearman rank correlations were performed**.** For both **C** and **D**, each data point represents a brain connection. The black line represents a least-squares linear regression line. *df*, degrees of freedom (2,695 data points displayed for each figure).

Connection length was defined as the shortest weighted path length between 2 brain regions in the healthy brain network. An example schematic of the shortest weighted path is shown in Figure 3A. For connections in the averaged healthy control brain, a one-way ANOVA was performed to assess differences in connection length for different connection subtypes: intramodular, intrahemispheric, interhemispheric, and corticostriatal. This was highly significant (F(3, 2691)= 739.23, *P* < 0.000; Figure 3B). Following this post-hoc test with the Tukey-Kramer test revealed clear step-wise differences in connection length across connection subtypes for healthy controls, such that all groups were significantly different from each other (*P* < 0.000; Supplemental Table 4). Cortico-striatal connections were the longest, followed by interhemispheric, intrahemispheric, and intramodular connections. See Supplemental Figure 3 for histograms of connection lengths for each connection type.

### Length of white matter connection in healthy controls determines cross-sectional and longitudinal connection atrophy in preHD.

Next, we investigated the relationship between connection length and its vulnerability to atrophy. For each connection within each subtype, connection strength and rate of change in connection strength over 24 months for preHD were normalized for preHD relative to controls. These were then transformed to give positive atrophy and rate of atrophy measures, where higher scores represent greater connection atrophy. The atrophy score was used in the cross-sectional analysis, while the rate of atrophy score was used in the longitudinal analysis. These scores were then averaged across the preHD group and correlated with the connection length for the same connection in the average healthy control group. Positive correlation between connection length and connection atrophy across subtypes collectively was seen both cross-sectionally (ρ = 0.54, *P* < 0.000, degrees of freedom [*df*] = 2,693) and longitudinally (ρ = 0.38, *P* < 0.000, *df* = 2,693; Figure 3, C and D). The cortico-striatal connections, the longest connections, showed both the greatest atrophy and the greatest rate of change, followed by interhemispheric connections, intrahemispheric connections, and intramodular connections, confirming our hypothesis that their vulnerability is related to their length. Given the differences in age and sex between cohorts (Supplemental Tables 5 and 6), all analyses were performed on residuals of connection strength after regressing out age and sex covariates for each participant. In order to establish whether our findings were influenced by study site, a split-site analysis was carried out. Sites were split based on type of MRI scanner: Leiden-Vancouver (Philips) cross-sectionally (ρ = 0.41, *P* < 0.000, *df* = 2,693) and longitudinally (ρ = 0.30, *P* < 0.000, *df* = 2,693), and London-Paris (Seimens) cross-sectionally (ρ = 0.48, *P* < 0.000, *df* = 2,693) and longitudinally (ρ = 0.34, *P* < 0.000, *df* = 2,693) (Supplemental Figures 4 and 5). Thus, results were consistent with the analysis across 4 sites, suggesting measures are robust across sites.

### White matter connection subtypes are associated with global cognitive performance.

Our next aim was to investigate the pathophysiological relevance of white matter connection loss with respect to connection subtype. We used a global cognitive composite score (16), as this encompasses many of the cognitive tests that have been shown to be sensitive in HD (17). Association between connection strength and cognition was assessed by the main effect of global cognitive composite score at baseline for an LMER. Age, site, education, CAG, and time-by-CAG interaction were included as covariates.

Significant (*q* < 0.05) positive association was seen between connection strength and global cognitive composite score for the interhemispheric connection between the left and the right motor-occipital-parietal modules. Intramodular left fronto-cingulate connection strength also showed significant (*q* < 0.05) positive association with global cognitive composite score. Only *P* < 0.05 positive association was seen for cortico-striatal and intrahemispheric connections and global cognitive composite score (Table 3). See Supplemental Table 7 for VCP results.

Longitudinally, no (*q* < 0.05) significant associations were seen between connection strength and global cognitive composite score. Negative association (*P* < 0.05) was seen for the connection strength in the interhemispheric connections between the left temporal and right temporal modules and the interaction between global cognitive composite and follow-up; similarly, negative association (*P* < 0.05) was seen for intramodular left fronto-cingulate connection strength and the interaction between global cognitive composite and followup (Table 4, Supplemental Table 8, Supplemental Table 9). Our results therefore provide a link between connectivity loss and global cognitive impairment and suggest that loss of interhemispheric and intramodular connectivity, which seems to occur later in preHD, is the main driver of global cognitive impairment.

## Discussion

**Figure 4:**
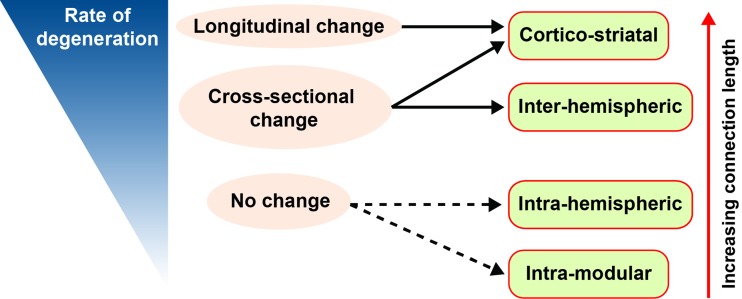
Schematic showing empirically determined hierarchy of white matter connection vulnerability in premanifest Huntington’s disease. Connections with the largest shortest weighted path length have a higher rate of degeneration and show significant (*q* < 0.05) longitudinal change. Both cortico-striatal and interhemispheric connections showed significant (*q* < 0.05) cross-sectional change, while neither intrahemispheric or intramodular connections showed significant (*q* < 0.05) group differences. Red arrow indicates increasing path length with respect to connection type. Blue wedge indicates rate of degeneration: slow rate represented by thin wedge and fast rate represented by thick wedge. Full black arrow represents *q* < 0.05; dashed black arrow represents *P* < 0.05.

In this study, we reveal a hierarchy of white matter connection vulnerability in preHD relative to controls, where greatest loss in connection strength is seen in cortico-striatal connections, followed by interhemispheric, intrahemispheric, and intramodular connections. The topological length of white matter connections determined this hierarchy with evidence of a positive association between topological white matter connection length and both cross-sectional and longitudinal loss of connection strength over 24 months (Figure 4). Furthermore, the pathophysiological relevance of these changes was demonstrated by correlations with global cognition.

This is the first study in HD to identify a temporal neuroanatomical pattern of white matter connection vulnerability. In preHD, reduced connection strength is seen in cortico-striatal connections both longitudinally and cross-sectionally compared with controls. While longitudinal change is seen in predominantly posterior cortico-striatal connections, cross-sectional differences were seen in all cortico-striatal connections. Interhemispheric connections also show significant cross-sectional reductions in preHD compared with controls between the posterior motor-occipital parietal modules and in their connections with the anterior fronto-cingulate modules. These findings are consistent with the cortico-striatal (18–20), corpus callosum (5, 21–23), and posterior (3, 6) white matter changes that have been identified in preHD.

Striatal pathology in HD occurs along a caudo-rostral, medio-lateral, dorso-ventral gradient (24), and thus, the posterior vulnerability of cortico-striatal white matter connections is in keeping with striatal pathology. Of the interhemispheric connections that did not differ cross-sectionally, all were connected to the temporal modules, again following the medial-lateral gradient of striatal pathology in HD (24) and temporal sparing that has been demonstrated previously (25). Cortical regions that show either vulnerability or resilience in preHD should be taken into account when assessing the distribution of ASOs in the cortex, such that higher concentrations in posterior cortical regions may be more beneficial than an equal distribution throughout the cortex.

Only one other study has investigated longitudinal change in brain networks in preHD (26). However, only regional changes in graph theory metrics were investigated. Changes in white matter connections were not examined; therefore, it is difficult to draw comparative conclusions regarding the longitudinal cortical-striatal connectivity changes we demonstrate here. In the aforementioned study, no group differences were seen at baseline, and longitudinal changes were only seen in the left orbitofrontal cortex and left paracentral lobule, with no regional changes seen in the striatum. These findings are not consistent with our previous cross-sectional structural connectivity study in HD (20). This may be due to the very small sample size and the fact a streamline filtering algorithm, such as SIFT (27) or SIFT2 (28), was not applied following diffusion tractography.

Prion-like spread is the spread of pathogenic proteins throughout the brain from cell to cell and is seen in a number of neurodegenerative diseases (29, 30). Systems level evidence for this has been shown in fronto-temporal dementia (FTD) (31, 32), where the distance from the brain region showing the earliest atrophy to other brain regions in the brain network predicts atrophy. However, in our previous study, we were unable to replicate this finding in an HD cohort (20). While we did find selective vulnerability of highly connected hub or “rich club” brain regions, this finding is generalizable across a number of brain disorders (33) and may be due to the high metabolic demands (34, 35) of hub brain regions, as opposed to a disease-specific mechanism such as prion-like spread. Indeed, hub brain regions with long-range connections have genetic transcription profiles enriched for oxidative metabolism and mitochondria (36, 37).

In this study, we provide evidence for the underlying cause behind the hierarchical loss of connectivity by showing a direct relationship between topological connection length and rate of atrophy over 24 months. As longer white matter connections are more metabolically active (11), the fact that these show the greatest rate of atrophy in preHD suggests the metabolic disturbances in HD (38–40) may be driving the degenerative process. However, we acknowledge that the data we present demonstrates association between topological connection length and connection atrophy and not causality. Furthermore, we do not assess the relationship between these white matter changes and either metabolism or mitochondrial function. Additionally, while path length is a topological measure of white matter length between 2 brain regions (41), the relationship of this measure to biological white matter tract length has not been established.

Cortico-striatal VCP and connectome cross-sectional analyses were in agreement; however, the longitudinal results differ slightly. VCPs show significant difference in the fronto-striatal connections, whereas the connectome analysis did not. This is likely due to methodological differences between each technique. VCPs calculate the number of voxels in the striatum that connect to a cortical region and are normalized by striatal and cortical volumes. The connectome analysis is based on the strength of connections between the striatum and cortical regions. Volume normalization was not performed in the connectome analysis, as we have shown previously that it can lead to spurious results (20).

Despite the fact that cortico-striatal connections show the largest change in connection strength, they did not show a strong association with the global composite score. Interhemispheric and intramodular connections show the strongest relationship with global composite cognitive score, showing FDR-corrected significance cross-sectionally and uncorrected significance longitudinal. The absence of a strong relationship between cortical-striatal connection strength and global cognition may be the reason why there is relatively little longitudinal change in cognitive performance in preHD over 24 months (17), as our results suggest degeneration of interhemispheric and intramodular connections is slower and occurs after cortico-striatal connection loss. Alternatively, by using a modular approach in order to simplify the interpretation of large numbers of brain connections, summing connections from multiple regions may result in loss of associations between cognitive variables and specific cortico-striatal connections that would otherwise be detectable.

In this study, we chose to focus on the caudate and putamen subcortical structures. This was based on observations from our cross-sectional structural connectivity study (20) and from the earlier Track-HD studies (3, 42) that show the caudate and putamen are the subcortical structures most affected in preHD both in terms of gray matter volume and white matter connections. While some studies have shown changes in the thalamus, globus pallidus, and nucleus accumbens in preHD, these tend to occur in preHD participants closer to disease onset (6, 43). Furthermore, automatic segmentation of globus pallidus and nucleus accumbens is not sufficiently reliable (44). Loss of white matter connections within the striatum was not examined, as our previous cross-sectional study did not show loss of these connections in preHD relative to controls (20).

### Conclusion.

Topological length of white matter connections predicts their rate of atrophy in preHD; this results in a hierarchy of vulnerability where cortico-striatal connections are most affected, followed by interhemispheric, intrahemispheric, and intramodular connections. This demonstrates a new principle underlying neurodegeneration in HD, whereby the brain connections with the greatest topological length are the first to suffer damage that can account for the stereotyped pattern of white matter loss observed in preHD.

## Methods

### Cohort

The cohort included preHD gene carriers and control participants from the Track-On HD study (16), followed up at 3 time points over 24 months at 4 sites (London, England; Leiden, Netherlands; Paris, France; and Vancouver, Canada). The total number of participants at each year was as follows: year 1 (72 preHD, 85 controls), year 2 (82 preHD, 87 controls) and year 3 (80 preHD, 80 controls). Track-On is an extension of the Track-HD (45) study, but with only preHD and control participants carried over (early HD participants from Track-HD were excluded). Of the participants included, 31 preHD and 29 controls had participated previously in Track-HD (45). The preHD participants required a disease burden score (DBS) > 250 (46), on the basis of their medical records at the time of assessment. Controls were selected from the spouses or partners of preHD individuals or were gene-negative siblings, to ensure consistency of environments. For this study, we excluded participants who had manifest disease at baseline, were left handed or ambidextrous, or had poor quality diffusion-weighted imaging (DWI) data, as defined by visual quality control.

With respect to missing data, 56 preHD and 65 controls had data at 3 time points, 28 premanifest and 24 controls had data at 2-time points, and 10 preHD and 9 controls had data at one time point. An LMER was used to account for missing data (see Statistics section), such that all data were included in the LMER analysis. See Supplemental Table 5 for baseline demographic information. For the rate of connection atrophy vs. shortest weighted path length (longitudinal) analysis, only preHD participants who had diffusion data from all 3 time points were included (56 preHD, 65 controls). See Supplemental Table 6 for demographic information of this cohort.

### MRI Acquisition

Data were acquired on 4 different 3T MRI scanners from 2 different manufacturers (Philips Achieva at Leiden and Vancouver and Siemens TIM Trio at London and Paris), both using a 12-channel head coil. T1-weighted image volumes were acquired using a 3-D MPRAGE acquisition sequence with the following imaging parameters: repetition time (RT) = 2,200 ms (Siemens [S])/ 7.7 ms (Philips [P]), time of echo (TE) = 2.2 ms (S)/3.5 ms (P), fractional anisotropy (FA) = 10◦ (S)/8◦(P), field of view (FOV) = 28 cm (S)/ 24 cm (P), matrix size 256 × 256 (S) / 224 × 224 (P), and sagittal slices 208 (S)/164 (P) to cover the entire brain with a slice thickness of 1.0 mm with no gap.

Diffusion-weighted images were acquired with 42 unique gradient directions (*b* = 1,000 sec/mm^2^). Eight images with no diffusion weighting (*b* = 0 sec/mm^2^) and one image with no diffusion weighting (*b* = 0 sec/mm^2^) were acquired from the Siemens and Philips scanners, respectively. For the Siemens scanners, TE = 88 ms and RT = 13 s; for the Phillips scanners, TE = 56 ms and RT = 11 s. Voxel size for the Siemens scanners was 2 × 2 × 2 mm, and voxel size for the Phillips scanners was 1.96 × 1.96 × 2 mm. Seventy-five slices were collected for each diffusion-weighted and non–diffusion-weighted volume. Scanning time was approximately 12 minutes for T1-weighted and 10 minutes for diffusion-weighted acquisitions.

### MRI data analysis

#### Structural MRI data.

Cortical and subcortical ROIs were generated by segmenting a T1-weighted image using FreeSurfer (47). These included 70 cortical regions and 4 subcortical regions (caudate and putamen bilaterally). We chose to focus on the caudate and putamen subcortical structures based on observations from our cross-sectional structural connectivity study (20) and from the earlier Track-HD studies (3, 42) that show the caudate and putamen are the subcortical structures most affected in preHD both in terms of gray matter volume and white matter connections. While some studies have shown changes in the thalamus, globus pallidus, and nucleus accumbens, in preHD, these tend to occur in preHD participants closer to disease onset (6, 43). Furthermore, automatic segmentation of globus pallidus, nucleus accumbens, and amygdala are not sufficiently reliable (44). The cerebellum was not included, as associated diffusion data was incomplete.

### Diffusion tensor imaging data

#### Data preprocessing.

For the diffusion data, the b = 0 image was used to generate a brain mask using FSL’s brain extraction tool (48). Eddy current correction was used to align the diffusion-weighted volumes to the first b=0 image and the gradient directions updated to reflect the changes to the image orientations. Finally, diffusion tensor metrics were calculated, and constrained spherical deconvolution (CSD) was applied to the data as implemented in MRtrix (49). FreeSufer ROIs were warped into diffusion space by mapping between the T1-weighted image and fractional anisotropy (FA) map using NiftyReg (50) and applying the resulting warp to each of the ROIs. A foreground mask was generated by combining FreeSurfer segmentations of white matter and gray matter. For the VCPs, 2 foreground masks were generated — one for the left hemisphere and the other for the right hemisphere — allowing investigation of intrahemispheric connectivity for each subcortical region.

#### Diffusion tractography.

Whole brain probabilistic tractography was performed using the iFOD2 algorithm in MRtrix (49). Specifically, 5 million streamlines were randomly seeded throughout the white matter, in all foreground voxels where FA > 0.2. Streamlines were terminated when they either reached gray matter or exited the foreground mask. The spherical deconvolution informed filtering of tractograms (SIFT2) algorithm (28) was used to reduce biases. The resulting set of streamlines was used to construct the structural brain network.

For the VCPs, 5,000 streamlines were seeded for each voxel within the subcortical ROIs and terminated when they reached the cortical mask or exited the hemisphere mask. The probability of connectivity between every seed voxel and every target region was established for each subject, and the data were stored as individual subject connectivity probability maps. The connectivity maps were first binarized such that any voxel within the subcortical ROI with at least 1% of streamlines reaching a given cortical target was regarded as being connected to that target. The number of voxels connected to the cortical target were then calculated and normalized by the sum of the volumes of the corresponding subcortical ROI and cortical target, providing a normalized estimate of the volume of the region connected to the target. The procedure was repeated for all cortical targets, resulting in a vector describing the connectivity between the striatum and cortex for each subject.

#### Construction of structural connectivity matrices.

For structural connectivity matrices, ROIs were defined as connected if a fibre originated in ROI 1 and terminated in ROI 2. Structural connections were weighted by streamline count and a cross-sectional area multiplier, as implemented in SIFT2 (28). Connections were then combined into 76 × 76 undirected and weighted matrices. As there is no consensus in the literature regarding the optimal graph thresholding strategy (51) and results can vary widely based on the chosen approach (52), SIFT2 was our preferred method of bias correction. Indeed, the creators of SIFT2 argue against the use of graph theory thresholding, as it introduces an arbitrary threshold value (53). SIFT2 was chosen in preference to SIFT, as it requires much less processing time and retains the full connectome (28).

### Modular organization

The cortex was split into distinct modules using the community Louvain algorithm (13) as implemented in the brain connectivity toolbox (BCT) (54) version 2016-01-16. This was performed on a group connectivity matrix created by averaging connectivity matrices across all participants. As module assignment can vary between runs of the algorithm, the algorithm was run 1,000 times and the most common assignment chosen using a consensus approach, also implemented in BCT. We chose the default resolution parameter γ = 1, as this represents classic modularity. This resulted in a module partition number of 6. We also generated module partition numbers of 4 and 8 using γ = 0.6 and γ = 1.7, respectively, to ensure this parameter did not affect our key results (Supplemental Figure 1 and Supplemental Figure 2).

### Statistics

All statistical analysis was performed in MATLAB v8.3. LMER was used as implemented in the MATLAB statistics and machine learning toolbox with the fitlme() function. An LMER was used, as it provides unbiased estimates under the assumption that the missing data is ignorable. LMER accounts for the dependence due to repeated measures, and our application was similar to a previous approach used in a longitudinal HD imaging study (55). Suppose that Y*_ij_* is the connection strength for the *i^th^* participant ( *i = 1, ... , N*) at the *j^th^* time point (*j = 1, ... , n_i_*), with time metric *t_ij_ = visit_ij_ – 1*, so that *t_i1_ = 0* . Furthermore, *group_i_* is a dummy variable taking the value of 0 if a participant is in the control group and the value of 1 if preHD. Then the LMER model was shown as Equation 1 below:

*Y_ij_* = *α* + *βt_ij_* + *γ (group_i_)* + *δ (group_i_) (t_ij_)* + *θX_i_* + *a_i_* + *b_i_ t_ij_* + *e_ij_*

where Greek letters denote fixed effects; *α* is the control group mean at the first visit, *β* is the control group linear slope, *γ* is the mean difference among the preHD and control groups at the first visit (difference of intercepts), *δ* is the slope difference among the groups (rate of change difference), *X_i_* is the matrix of covariates (age, sex, site, education) with associated regression coefficient vector *θ*; *a_i_* and *b_i_* are random effects (random intercepts and slopes), and *e_ij_* is random error. Maximum likelihood methods are used for estimation under the assumption that the random effects have a joint-normal distribution with zero-means and nonsingular covariance matrix, and the random error is normally distributed with zero-mean and constant nonzero variance. The objects of inference in Equation 1 were *γ* and *δ* , with the former being the initial cross-sectional mean difference among the groups adjusting for the covariates and the latter being the group difference in the rate of change (slope difference) adjusting for the covariates. The null hypothesis of interest were *H_0_:* *γ = 0* (no initial mean group difference) and *H_0_:* *δ = 0* (no group difference in rate of change), which were tested with the *z*-values of *z =* *γ ^/SE(γ ^)* and *z =* *δ ^ / SE (δ ^)*. FDR was applied for testing multiple connections in each connection subgroup (56).

A similar model as equation 1 was used to explore the association between connection strength and cognition in preHD, where the continuous baseline cognitive variable (*c_i_*) replaced the dummy group variable in the LMER model (i.e., *γc_i_ + δc_i_t_ij_*). A global cognitive composite score (16) was chosen as the cognitive variable of interest, as this encompasses many of the cognitive tests that have been shown to be sensitive in HD (17) and, thus, provides an overall indicator of cognitive function at the start of the study.

### Connection subtypes

Connections were classed as cortico-striatal, defined as the sum of connection weights between the striatum (caudate and putamen) and cortical modules; interhemispheric, defined as connections between left and right cortical modules; intrahemispheric, defined as connections between cortical modules in the left and right hemispheres separately; and intramodular, defined as the sum of connection weights with cortical modules.

### Shortest weighted path length and connection subtype in healthy controls

Connection length, defined as shortest weighted path length, was computed for every pair of brain regions in the averaged healthy control brain network using the BCT (54). An illustration of the shortest weighted path length from an example network is shown in Figure 3A. First, the weighted connectivity matrix was converted to a connection-lengths matrix where higher weights are interpreted as shorter lengths. This connection-lengths matrix is defined as the inverse of the weighted connectivity matrix. Dijkstra’s algorithm (57) was then used to calculate the shortest weighted path between each pair of brain regions. The relationship between connection length and connection type was then investigated using a one-way ANOVA. Post-hoc analysis was then performed using the Tukey-Kramer test (58) in order to investigate if the connection length for each connection subtype was significantly different from the connection length of other connection subtypes.

### Shortest weight path length in healthy controls and rate of connection atrophy in preHD

Spearman correlations were performed in order to assess the relationship between shortest weighted path length of a connection in healthy controls and connection atrophy in preHD both cross-sectionally and longitudinally. For each participant, age and sex where regressed out from connection strength measures and subsequent analysis was performed using the resulting residuals. In order to establish whether our findings were influenced by study site, a split-site analysis was carried out. Sites were split based on type of MRI scanner; cross-sectional analysis Leiden-Vancouver (Philips) 29 preHD and 36 controls, London-Paris (Siemens) 43 preHD and 49 controls, longitudinal analysis Leiden-Vancouver (Philips) 18 preHD and 25 controls, London-Paris (Siemens) 38 preHD and 40 controls.

### Cross-sectional analysis

For the cross-sectional analysis, a Z-score was calculated as follows:

*Z_c_(i) = C_k_(i) –* μ *(C_h_[i]) / σ (C_h_[i])*

In this equation, *i* is the connection, *k* is preHD, *h* is healthy controls, *C* is connection strength, μ is mean, and σ is standard deviation. This was then transformed in order to produce positive atrophy measures between 0 and 1, using the following equation:

*Z_C–T_ (i) = 1 / e^–Z^_C_^(i)^ + e^+Z^_C_^(i)^*

This approach has been used previously to model Alzheimer’s disease atrophy based on properties of the healthy connectome (59). This resulted in a transformed Z-score for each connection for each preHD participant. An average was then calculated across the preHD group, resulting in a single transformed Z-score for each connection, and these were correlated with shortest weighted path length for each connection, calculated from an average control group.

### Longitudinal analysis

For each preHD participant and for each connection, a least squares line was fitted over the connection weights across time points and the rate of connection atrophy defined as the gradient of the least squares line. A Z-score was then calculated using the following equation:

*Z_R_ (i) = R_k_ (i) –* μ *(R_h_[i]) / σ (R_h_ [i])*

In this equation, *R* is the rate of change of connection strength. This was then transformed in order to produce positive rate of atrophy measures between 0 and 1, using the following equation:

*Z_R–T_ (i) = 1 / e^–Z^_R_^(i)^ + e^+Z^_R_(i)*

This resulted in a transformed Z-score of rate of connection atrophy for each connection for each preHD participant. An average was then calculated across the preHD group, resulting in a single transformed Z-score for each connection, and these were correlated with shortest weighted path length for each connection, calculated from an average control group.

### Study approval

Informed consent was obtained from each participant, and the study protocol was approved by the following local ethics committees. London, England: National Research Ethics Service Committee London, Queen Square. Leiden,Netherlands: De Commissie Medische Ethiek van het Leids Universitair Medisch Centrum. Paris, France: Comite De Protection Des Personnes Ile-de-France VI Pitie Salpetriere Hospital. Vancouver, Canada: The University of British Columbia Clinical Research Ethics Board.

## Author contributions

PM contributed to study design, analysis, and manuscript preparation. KKS contributed to study design, analysis, and manuscript review. SG contributed to study design and manuscript review. AR contributed to study design and manuscript review. MP contributed to manuscript review. JDL and JAM contributed to analysis and manuscript review. EJ contributed to analysis and manuscript review. AD, RACR, and BRL contributed to data collection and manuscript review. JCS contributed to study design and manuscript review. RIS contributed to study design and manuscript review. CAC contributed to study design and manuscript review. GR contributed to study design and manuscript review. SJT contributed to study design, data collection, and manuscript review.

## Supplementary Material

Supplemental data

## Figures and Tables

**Table 4:**
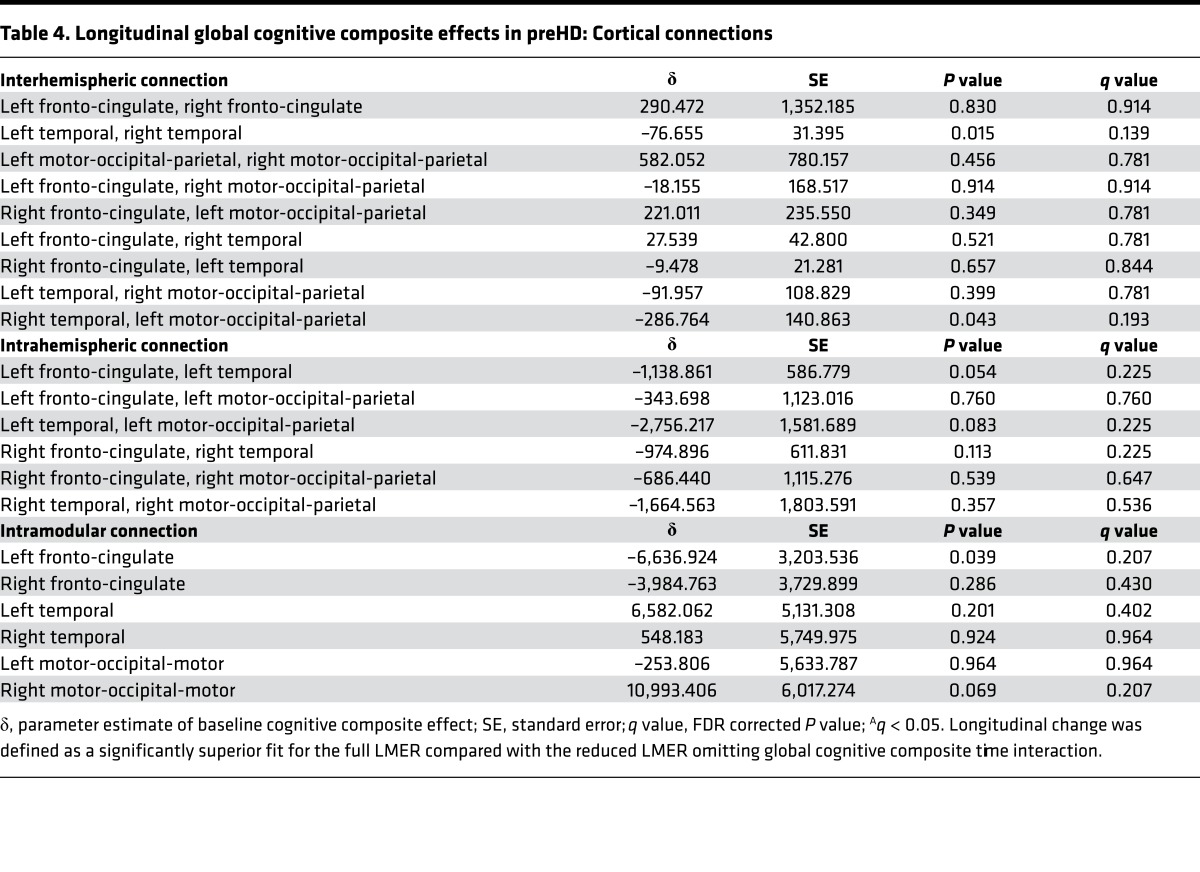
Longitudinal global cognitive composite effects in preHD: Cortical connections

**Table 3:**
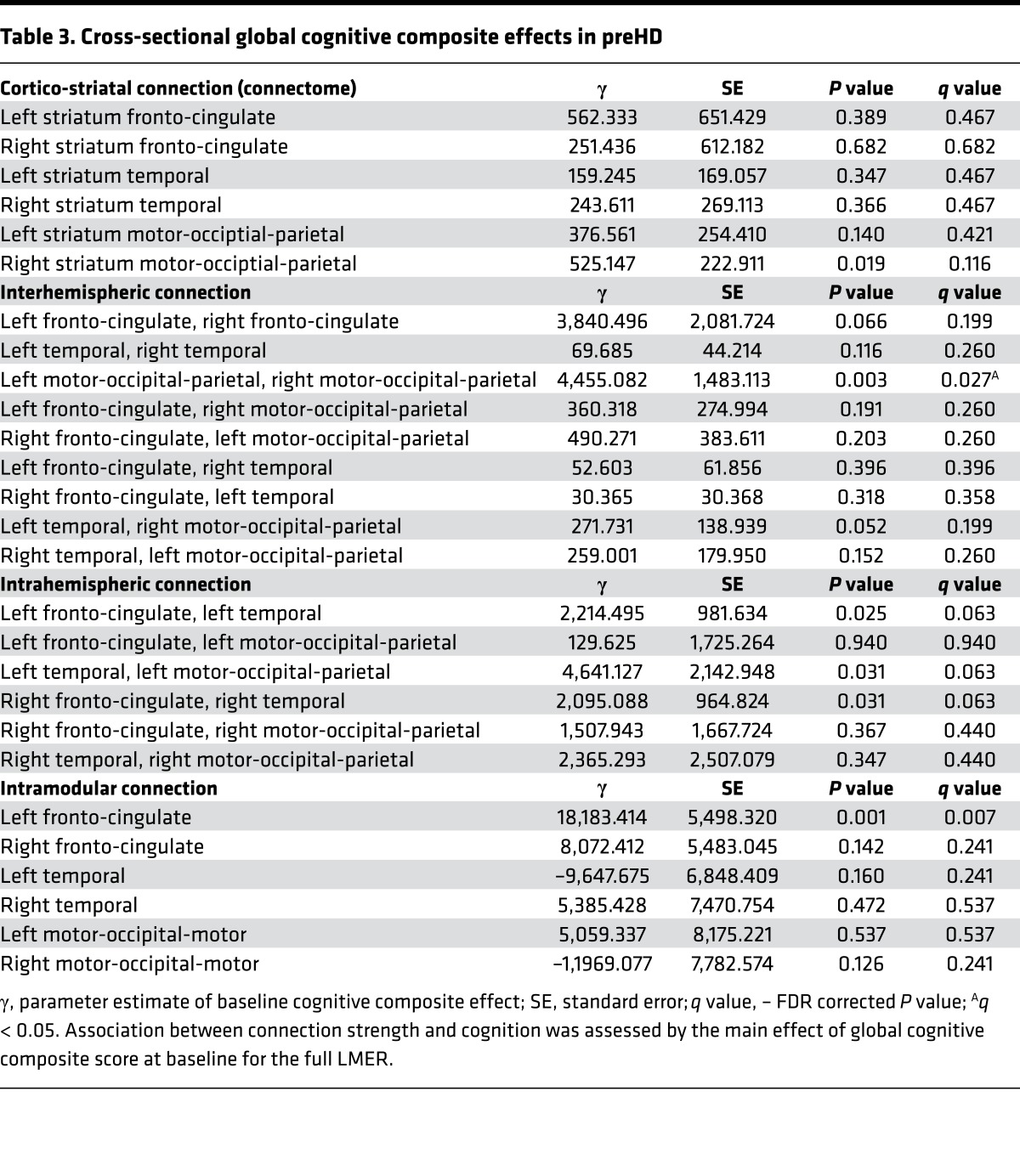
Cross-sectional global cognitive composite effects in preHD

**Table 2:**
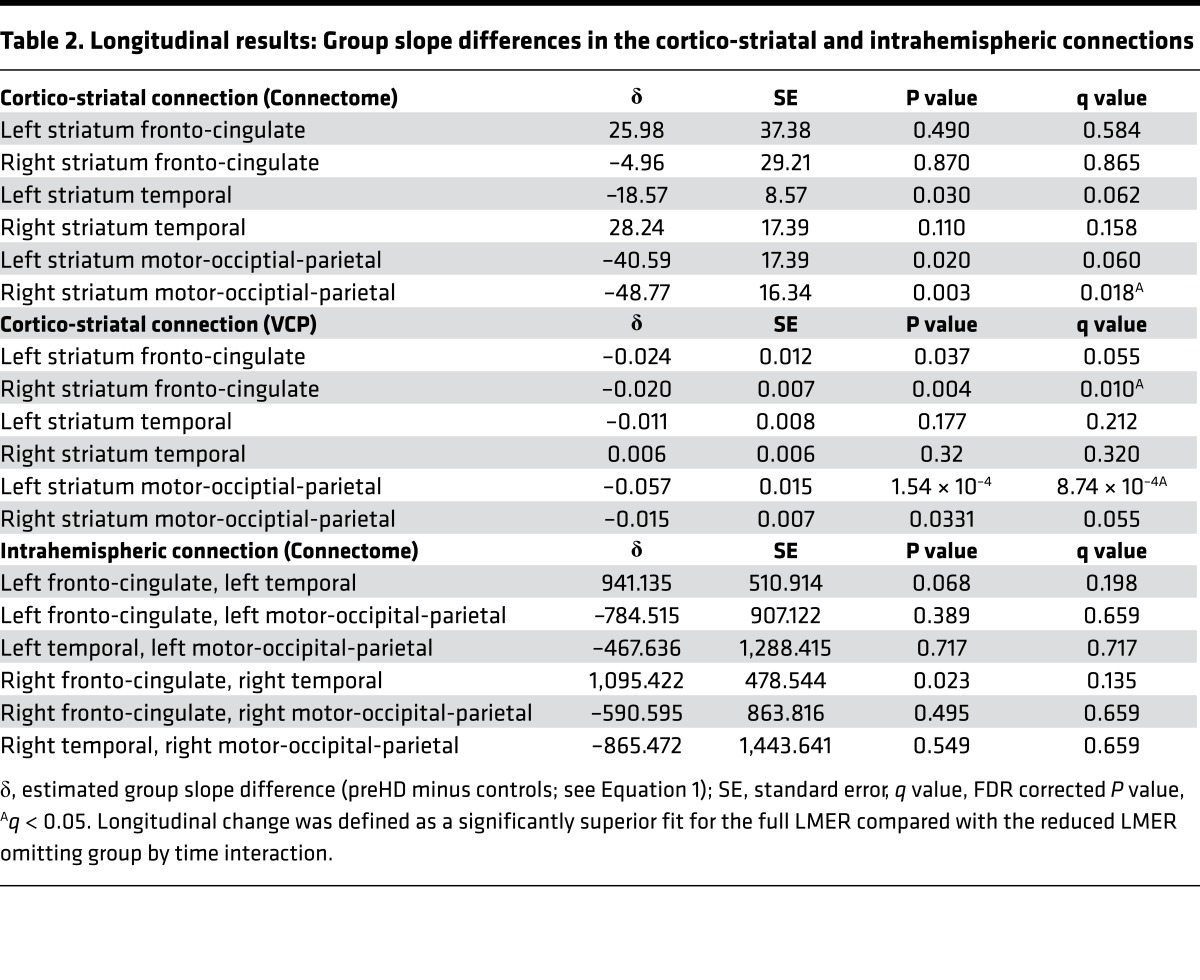
Longitudinal results: Group slope differences in the cortico-striatal and intrahemispheric connections

**Table 1:**
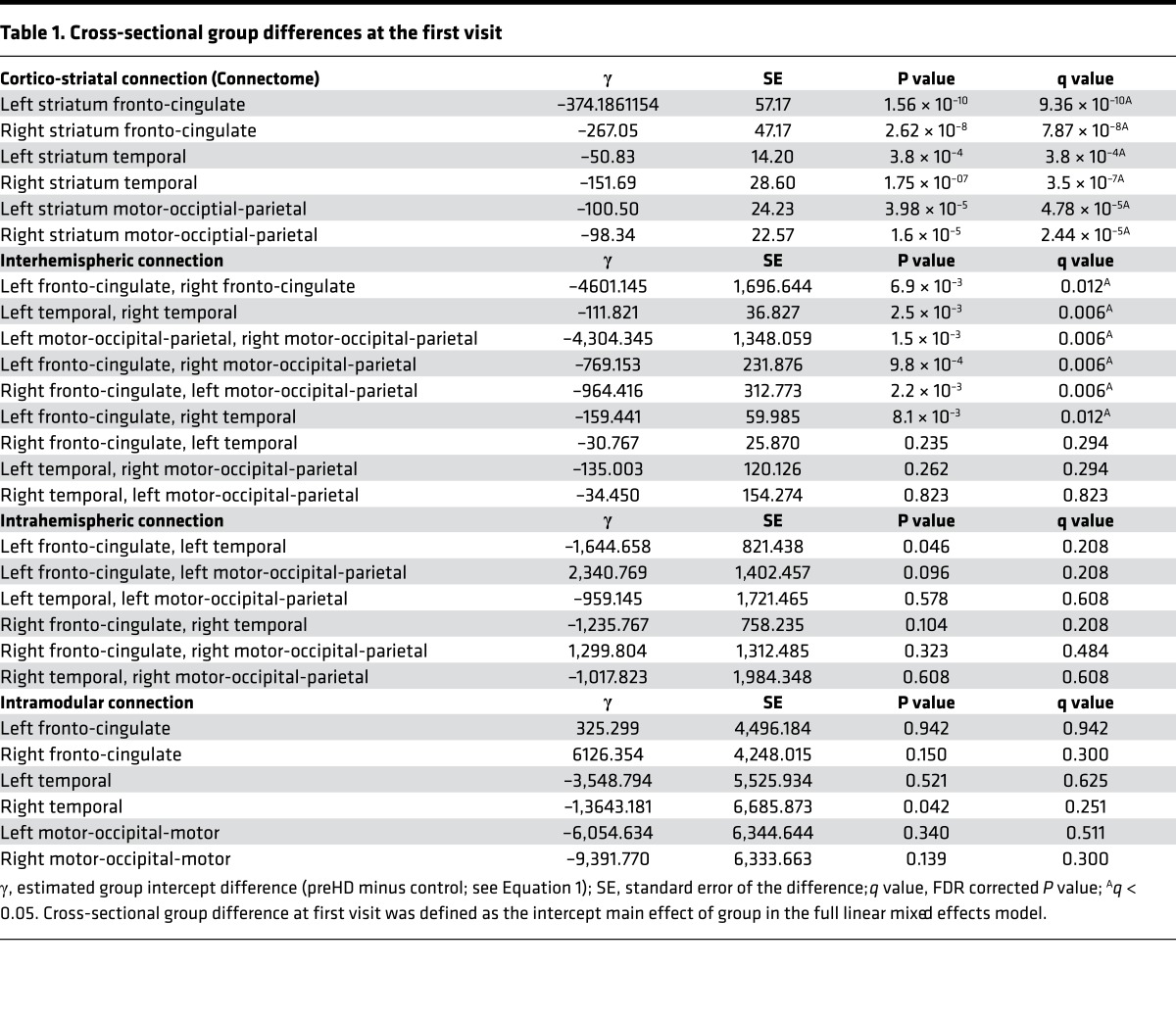
Cross-sectional group differences at the first visit
