# Supplementary material for: Topological length of white matter connections predicts their rate of atrophy in premanifest Huntington’s disease
Source: JCI Insight. 2017 Apr 20;2(8):e92641. doi: 10.1172/jci.insight.92641 (PMC5396531; doi:10.1172/jci.insight.92641)
Supplement: Supplemental data [file jciinsight-2-92641-s001.pdf]

**Supplemental Data**

**Supplemental Figure 1. Four cortical module analysis. Connection length correlates with rate of connection degeneration over 2 years in preHD. (A) Cross sectional analysis:** Z-scores, denoting loss of connection strength, were transformed into positive atrophy measures using a logistic transform. Average transformed connection strength Z-score for preHD participants was plotted against connection weighted path length for average control. Connections colour coded according to type**. (B) Longitudinal analysis:** Z-scores, denoting connection rate of atrophy over 3 time points, were transformed into a positive rate of atrophy measure using a logistic transform. Average transformed connection rate of change Z-score for preHD participants were plotted against connection weighted path length for average control and Spearman rank correlations performed**.** For both (A) and (B) each data point represents a brain connection. Intra-M – intra-modular (magneta), Intra-H –intra-hemispheric (green), Inter-H – inter-hemispheric (red), CS – cortico-striatal (blue). The black line represents a least squares linear regression line. rho = correlation, p = p-value, DF = degrees of freedom. Please note that data points and rho values are same for cortical module analyses 4, 6 and 8 as the module assignment does not alter the connection atrophy measures or the shortest weighted path length. Differing module partitions changes the assignment of connection type, specifically whether a connection is classed as intra-modular or inter/intra-hemispheric.

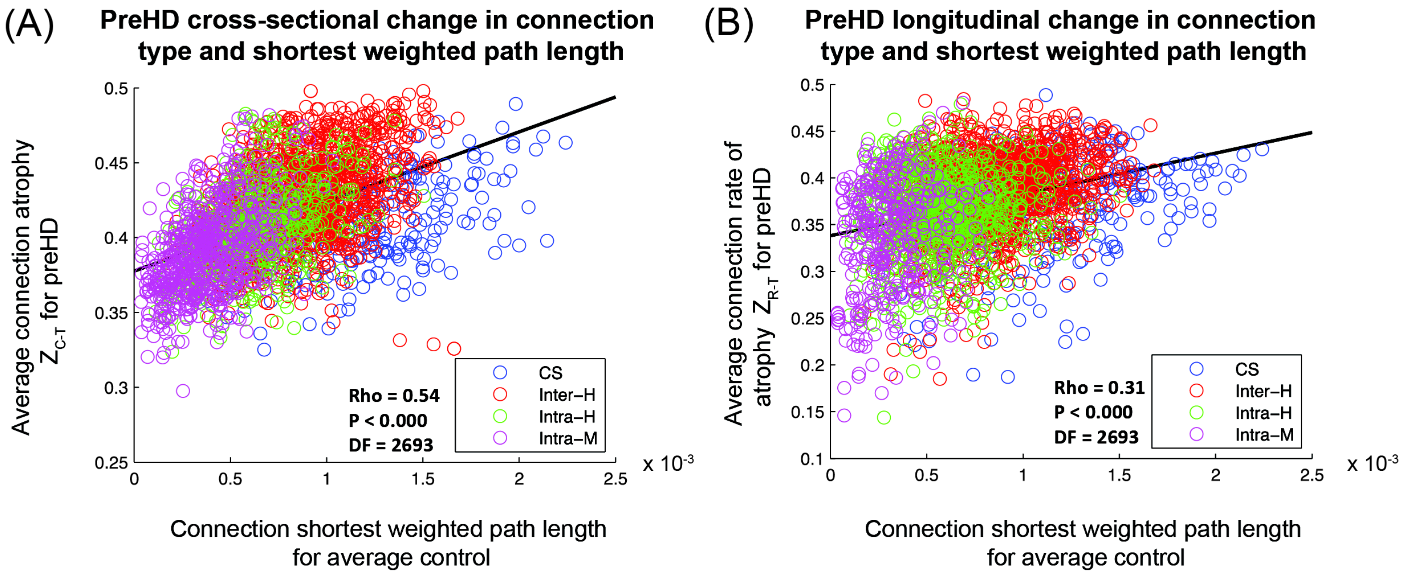

**Supplemental Figure 2. Eight cortical module analysis. Connection length correlates with rate of connection degeneration over 2 years in preHD. (A) Cross sectional analysis:** Z-scores, denoting loss of connection strength, were transformed into positive atrophy measures using a logistic transform. Average transformed connection strength Z-score for preHD participants was plotted against connection weighted path length for average control. Connections colour coded according to type**. (B) Longitudinal analysis:** Z-scores, denoting connection rate of atrophy over 3 time points, were transformed into a positive rate of atrophy measure using a logistic transform. Average transformed connection rate of change Z-score for preHD participants were plotted against connection weighted path length for average control and Spearman rank correlations performed**.** For both (A) and (B) each data point represents a brain connection. Intra-M – intra-modular (magneta), Intra-H –intra-hemispheric (green), Inter-H – inter-hemispheric (red), CS – cortico-striatal (blue). The black line represents a least squares linear regression line. rho = correlation, p = p-value, DF = degrees of freedom. Please note that data points and rho values are same for cortical module analyses 4, 6 and 8 as the module assignment does not alter the connection atrophy measures or the shortest weighted path length. Differing module partitions changes the assignment of connection type, specifically whether a connection is classed as intra-modular or inter/intra-hemispheric.

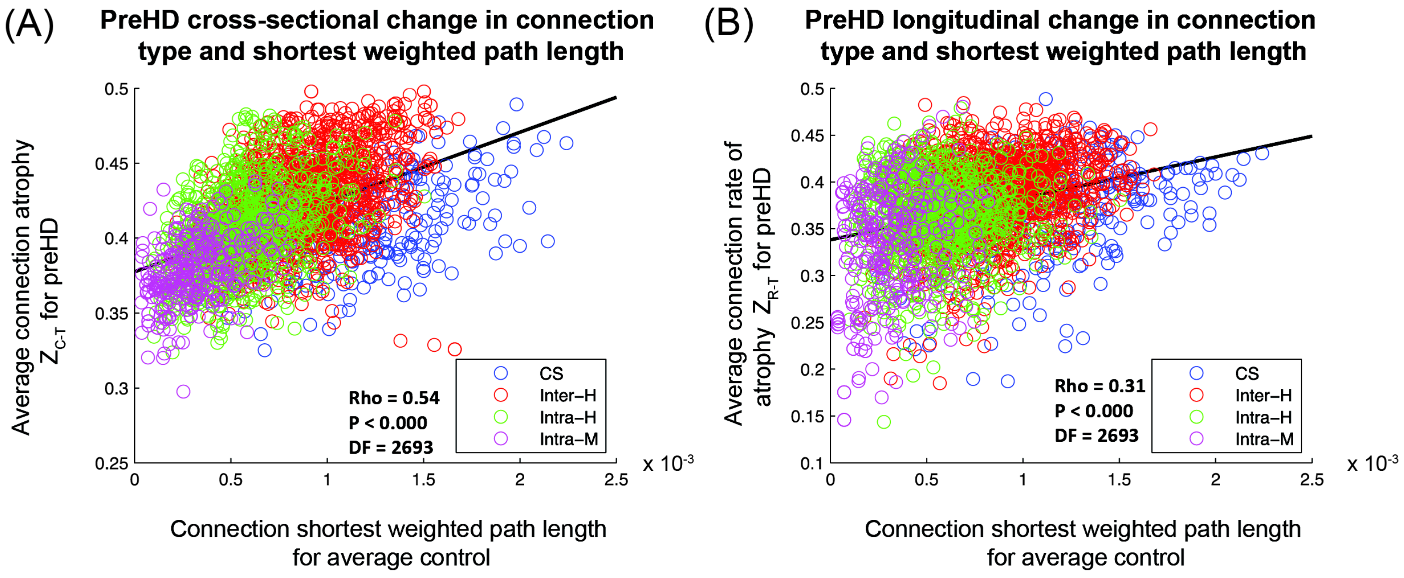

**Supplemental Figure 3. Histograms of *shortest weighted path length* for different classes of connection.** Intra-modular (magneta), Intra-hemispheric (green), Inter-hemispheric (red), Cortico-striatal (blue). Y-axis: frequency of connection length in a given interval (intervals generated automatically by MATLAB hist function in order to cover range of values in data). X-axis: *shortest weighted path length*

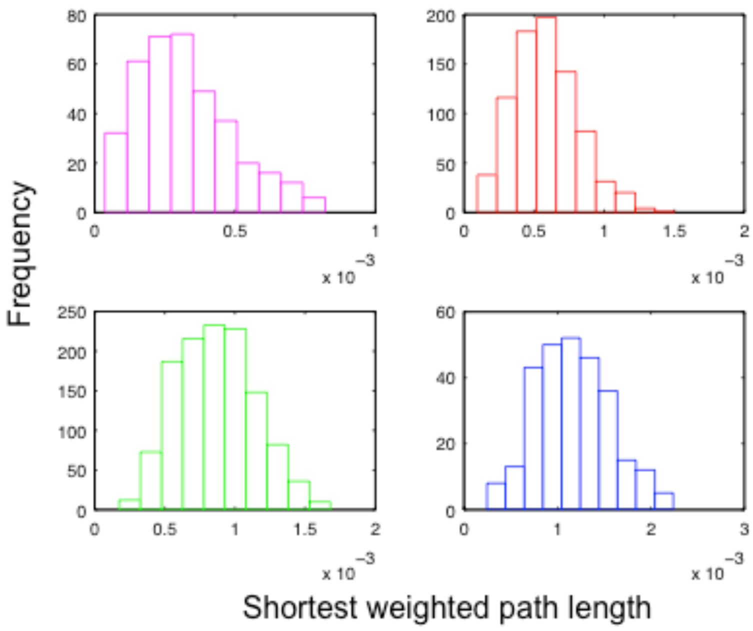

**Supplemental Figure 4**. **Split site Leiden-Vancouver analysis. Connection length correlates with rate of connection degeneration over 2 years in preHD. (A) Cross sectional analysis:** Z-scores, denoting loss of connection strength, were transformed into positive atrophy measures using a logistic transform. Average transformed connection strength Z-score for preHD participants was plotted against connection weighted path length for average control. Connections colour coded according to type**. (B) Longitudinal analysis:** Z-scores, denoting connection rate of atrophy over 3 time points, were transformed into a positive rate of atrophy measure using a logistic transform. Average transformed connection rate of change Z-score for preHD participants were plotted against connection weighted path length for average control and Spearman rank correlations performed**.** For both (A) and (B) each data point represents a brain connection. Intra-M – intra-modular (magneta), Intra-H –intra-hemispheric (green), Inter-H – inter-hemispheric (red), CS – cortico-striatal (blue). The black line represents a least squares linear regression line. rho = correlation, p = p-value, DF = degrees of freedom.

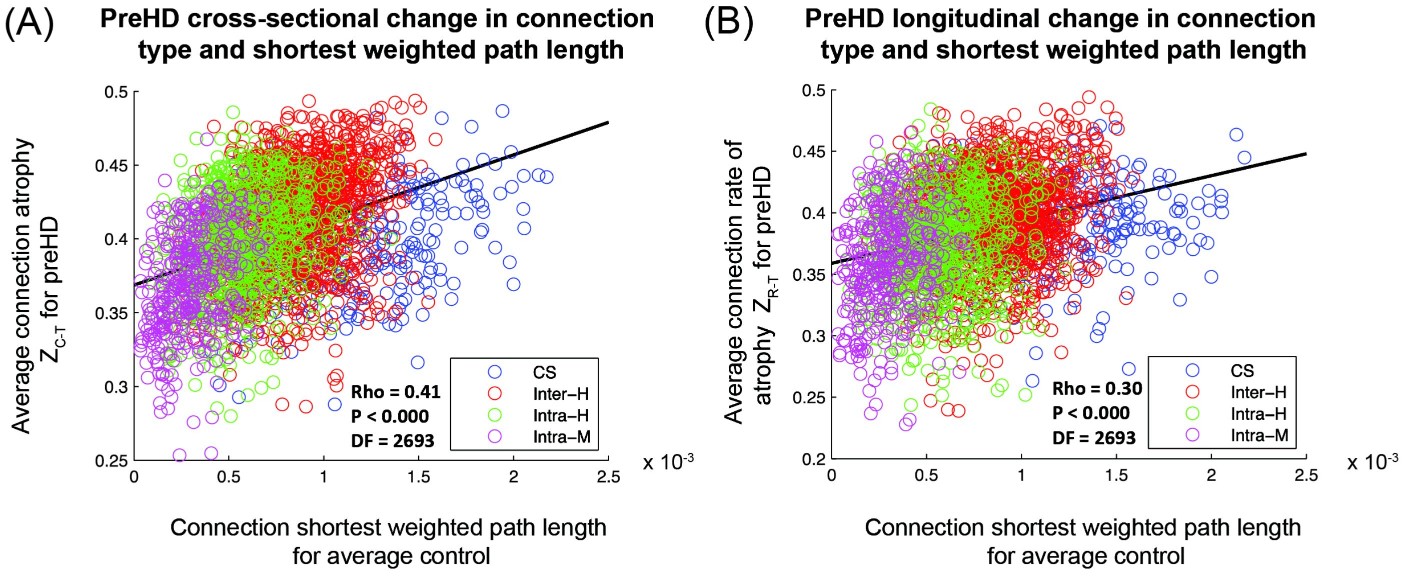

**Supplemental Figure 5**. **Split site London-Paris analysis. Connection length correlates with rate of connection degeneration over 2 years in preHD. (A) Cross sectional analysis:** Z-scores, denoting loss of connection strength, were transformed into positive atrophy measures using a logistic transform. Average transformed connection strength Z-score for preHD participants was plotted against connection weighted path length for average control. Connections colour coded according to type**. (B) Longitudinal analysis:** Z-scores, denoting connection rate of atrophy over 3 time points, were transformed into a positive rate of atrophy measure using a logistic transform.  Average transformed connection rate of change Z-score for preHD participants were plotted against connection weighted path length for average control and Spearman rank correlations performed**.** For both (A) and (B) each data point represents a brain connection. Intra-M – intra-modular (magneta), Intra-H –intra-hemispheric (green), Inter-H – inter-hemispheric (red), CS – cortico-striatal (blue). The black line represents a least squares linear regression line. rho = correlation, p = p-value, DF = degrees of freedom.

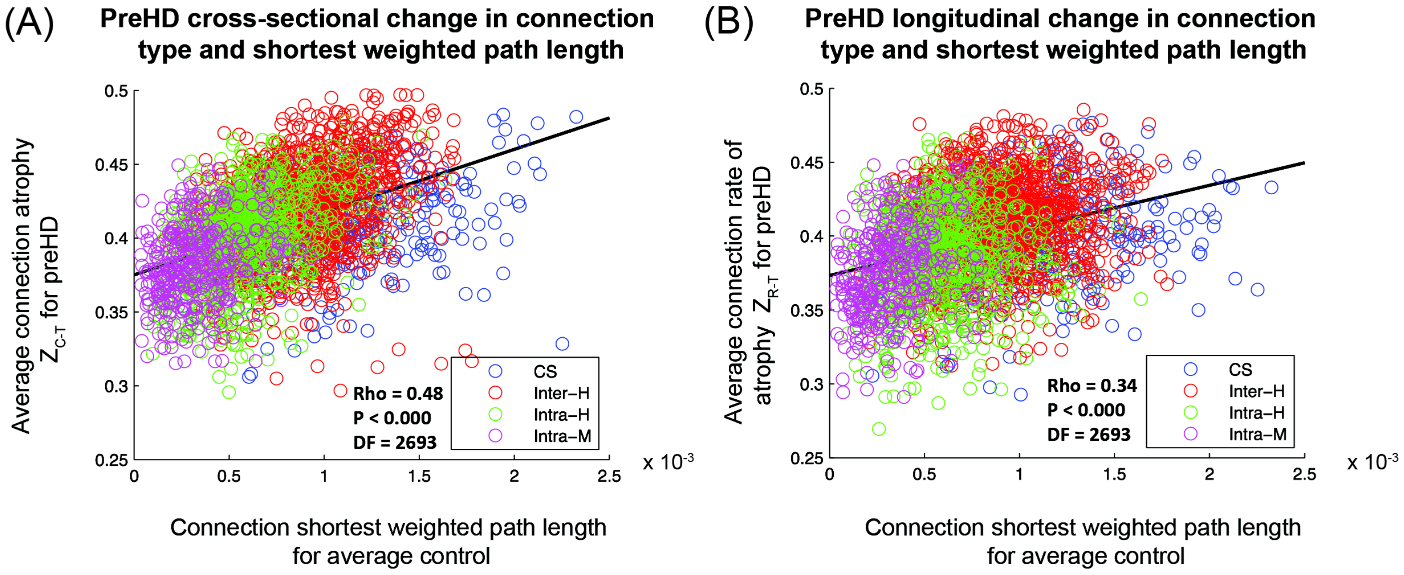

**Supplemental Table 1. Cortical module assignments**

| | Cortical Region | | Cortical Region |
|---|---|---|---|
| **Module 1** | L.caudalanteriorcingulate | **Module 2** | R.caudalanteriorcingulate |
| | L.caudalmiddlefrontal | | R.caudalmiddlefrontal |
| | L.lateralorbitofrontal | | R.lateralorbitofrontal |
| | L.medialorbitofrontal | | R.medialorbitofrontal |
| | L.parsopercularis | | R.parsopercularis |
| | L.parsorbitalis | | R.parsorbitalis |
| | L.parstriangularis | | R.parstriangularis |
| | L.rostralanteriorcingulate | | R.posteriorcingulate |
| | L.rostralmiddlefrontal | | R.rostralanteriorcingulate |
| | L.superiorfrontal | | R.rostralmiddlefrontal |
| | L.frontalpole | | R.superiorfrontal |
| | L.insula | | R.frontalpole |
| **Module 3** | L.bankssts | **Module 4** | R.hippocampus |
| | L.entorhinal | | R.bankssts |
| | L.fusiform | | R.entorhinal |
| | L.inferiortemporal | | R.fusiform |
| | L.middletemporal | | R.inferiortemporal |
| | L.parahippocampal | | R.middletemporal |
| | L.superiortemporal | | R.parahippocampal |
| | L.temporalpole | | R.superiortemporal |
| | L.transversetemporal | | R.temporalpole |
| | L.hippocampus | | R.transversetemporal |
| | | | R.insula |
| **Module 5** | L.cuneus | **Module 6** | R.cuneus |
| | L.inferiorparietal | | R.inferiorparietal |
| | L.isthmuscingulate | | R.isthmuscingulate |
| | L.lateraloccipital | | R.lateraloccipital |
| | L.lingual | | R.lingual |
| | L.paracentral | | R.paracentral |
| | L.pericalcarine | | R.pericalcarine |
| | L.postcentral | | R.postcentral |
| | L.posteriorcingulate | | R.precentral |
| | L.precentral | | R.precuneus |
| | L.precuneus | | R.superiorparietal |
| | L.superiorparietal | | R.supramarginal |
| | L.supramarginal | | |

**Supplemental Table 2. Cross-sectional mixed linear model results: Cortico-striatal (VCP) and intra-modular connections.** $\gamma$ – estimated group intercept difference (preHD minus control; see Equation 1), SE – standard error of the difference, p-value – probability value, q-value – FDR corrected p-value. Cross-sectional group difference at first visit was defined as the intercept main effect of group in the full linear mixed effects model.

| Cortico-striatal connection (VCP) | $\gamma$ | SE | p-value | q-value |
|---|---|---|---|---|
| Left striatum fronto-cingulate | -0.159 | 0.020 | $1.2 \times 10^{-14}$ | $7.23 \times 10^{-14}$ |
| Right striatum fronto-cingulate | -0.061 | 0.009 | $2.23 \times 10^{-10}$ | $6.69 \times 10^{-10}$ |
| Left striatum temporal | -0.046 | 0.014 | $1.1 \times 10^{-3}$ | $1.1 \times 10^{-3}$ |
| Right striatum temporal | -0.049 | 0.009 | $2.48 \times 10^{-7}$ | $4.95 \times 10^{-7}$ |
| Left striatum motor-occipital-parietal | -0.067 | 0.020 | $6.37 \times 10^{-4}$ | $7.64 \times 10^{-4}$ |
| Right striatum motor-occipital-parietal | -0.043 | 0.010 | $2.37 \times 10^{-5}$ | $3.55 \times 10^{-5}$ |
| **Intra-modular connection** | $\gamma$ | **SE** | **p-value** | **q-value** |
| left fronto-cingulate | 325.299 | 4496.184 | 0.942 | 0.942 |
| right fronto-cingulate | 6126.354 | 4248.015 | 0.150 | 0.300 |
| left temporal | -3548.794 | 5525.934 | 0.521 | 0.625 |
| right temporal | -13643.181 | 6685.873 | 0.042 | 0.251 |
| left motor-occipital-motor | -6054.634 | 6344.644 | 0.340 | 0.511 |
| right motor-occipital-motor | -9391.770 | 6333.663 | 0.139 | 0.300 |

**Supplemental Table 3. Longitudinal mixed linear model results: Inter-hemispheric and Intra-modular connections.** ***δ*** – parameter estimate, SE – standard error, p-value – probability valve, q-value – FDR corrected p-value. Longitudinal change was defined as a significantly superior fit for the full LMER compared to the reduced LMER omitting group * time interaction.

| Inter-hemispheric connection | $\delta$ | SE | p-value | q-value |
|---|---|---|---|---|
| left fronto-cingulate right fronto-cingulate | -8.548 | 1064.484 | 0.994 | 0.994 |
| left temporal right temporal | 7.415 | 28.474 | 0.795 | 0.946 |
| left motor-occipital-parietal right motor-occipital-parietal | 123.274 | 614.003 | 0.841 | 0.946 |
| left fronto-cingulate right motor-occipital-parietal | -112.598 | 143.000 | 0.431 | 0.647 |
| right fronto-cingulate left motor-occipital-parietal | -281.773 | 184.737 | 0.128 | 0.383 |
| left fronto-cingulate right temporal | -52.692 | 40.285 | 0.192 | 0.383 |
| right fronto-cingulate left temporal | -39.018 | 20.837 | 0.062 | 0.383 |
| left temporal right motor-occipital-parietal | -135.995 | 97.276 | 0.162 | 0.383 |
| right temporal left motor-occipital-parietal | -158.435 | 127.021 | 0.213 | 0.383 |
| **Intra-modular connection** | $\delta$ | **SE** | **p-value** | **q-value** |
| left fronto-cingulate | 3977.641 | 2647.570 | 0.134 | 0.401 |
| right fronto-cingulate | 2250.200 | 2861.948 | 0.432 | 0.520 |
| left temporal | 2698.917 | 4211.651 | 0.523 | 0.522 |
| right temporal | 3901.665 | 4978.346 | 0.434 | 0.520 |
| left motor-occipital-motor | 6834.844 | 4460.051 | 0.126 | 0.401 |
| right motor-occipital-motor | 4121.240 | 4923.315 | 0.403 | 0.520 |

**Supplemental Table 4. Tukey-Kramer post hoc analysis of differences in connection length between different connections types.** Intra-M –intra-modular, Intra-H –intra-hemispheric, Inter-H – inter-hemispheric, CS – cortico-striatal. CI – confidence interval.

| Group 1 | Group 2 | 95% lower CI | Mean difference | 95% CI upper | p-value |
|---|---|---|---|---|---|
| Intra-M | Intra-H | -0.000302067 | -0.000259613 | -0.000217159 | 3.77E-09 |
| Intra-M | Inter-H | -0.000582694 | -0.000542553 | -0.000502413 | 3.77E-09 |
| Intra-M | CS | -0.000897343 | -0.000843599 | -0.000789855 | 3.77E-09 |
| Intra-H | Inter-H | -0.000313728 | -0.00028294 | -0.000252152 | 3.77E-09 |
| Intra-H | CS | -0.000631157 | -0.000583986 | -0.000536816 | 3.77E-09 |
| Inter-H | CS | -0.000346146 | -0.000301046 | -0.000255947 | 3.77E-09 |

**Supplemental Table 5. Baseline demographic information**. SD = standard deviation, M = male, F = female, N = number. ISCED = International standard classification of education. CAG = CAG repeat expansion length, DBS = disease burden scale (Penney, et al., 1997)

| | Premanifest HD | Control | Statistical test | P-value |
|---|---|---|---|---|
| **N** | 72 | 85 | - | - |
| **Age (SD)** | 43.3 (9.2) | 48.8 (9.8) | T-test | 0.0004 |
| **Gender (M/F)** | 38/34 | 32/53 | Chi-square | 0.057 |
| **Study Site (N) (Leiden/London/Paris/Vancouver)** | 14/25/18/15 | 20/26/23/16 | Chi-square | 0.89 |
| **ISCED (2/3/4/5/6)** | 5/16/23/27/1 | 8/16/29/30/2 | Chi-square | 0.94 |
| **CAG (SD)** | 42 (2.3) | - | - | - |
| **DBS (SD)** | 317 (55) | - | - | - |

**Supplemental Table 6. Rate of connection atrophy longitudinal cohort.** SD = standard deviation, M = male, F = female, N = number. ISCED = International standard classification of education. CAG = CAG repeat expansion length, DBS = disease burden scale (Penney, et al., 1997)

| | Premanifest HD | Control | Statistical test | P-value |
|---|---|---|---|---|
| **N** | 56 | 65 | - | - |
| **Age (SD)** | 43.6 (9.3) | 49.2 (9.7) | T-test | 0.002 |
| **Gender (M/F)** | 30/26 | 24/41 | Chi-square | 0.066 |
| **Study Site (N) (Leiden/London/Paris/Vancouver)** | 8/22/16/10 | 15/20/20/10 | Chi-square | 0.57 |
| **ISCED (2/3/4/5/6)** | 5/13/14/23/1 | 5/16/22/20/2 | Chi-square | 0.74 |
| **CAG (SD)** | 42.8 (2.4) | - | - | - |
| **DBS (SD)** | 316.7 (58) | - | - | 10 |

**Supplemental Table 7. Cross-sectional global cognitive composite effects in preHD: Cortico-striatal connections (VCP).** $\gamma$ – parameter estimate of baseline cognitive composite effect, SE – standard error, p-value – probability valve, q-value – FDR corrected p-value. Association between connection strength and cognition was assessed by the main effect of global cognitive composite score at baseline for the full LMER.

| Cortico-striatal connection (VCP) | $\gamma$ | SE | P-value | q-value |
|---|---|---|---|---|
| Left striatum fronto-cingulate | 0.037 | 0.023 | 0.114 | 0.412 |
| Right striatum fronto-cingulate | 0.017 | 0.011 | 0.137 | 0.412 |
| Left striatum temporal | 0.009 | 0.014 | 0.528 | 0.723 |
| Right striatum temporal | 0.005 | 0.010 | 0.623 | 0.723 |
| Left striatum motor-occipital-parietal | 0.026 | 0.021 | 0.217 | 0.433 |
| Right striatum motor-occipital-parietal | -0.004 | 0.012 | 0.723 | 0.723 |

**Supplemental Table 8. Longitudinal global cognitive composite effects in preHD: Cortical connections.** $\delta$ – parameter estimate of baseline cognitive composite effect, SE – standard error, p-value – probability valve, q-value – FDR corrected p-value. Longitudinal change was defined as a significantly superior fit for the full LMER compared to the reduced LMER omitting global cognitive composite * time interaction.

| **Inter-hemispheric connection** | $\boldsymbol{\delta}$ | **SE** | **p-value** | **q-value** |
|---|---|---|---|---|
| left fronto-cingulate right fronto-cingulate | 290.472 | 1352.185 | 0.830 | 0.914 |
| left temporal right temporal | -76.655 | 31.395 | 0.015 | 0.139 |
| left motor-occipital-parietal right motor-occipital-parietal | 582.052 | 780.157 | 0.456 | 0.781 |
| left fronto-cingulate right motor-occipital-parietal | -18.155 | 168.517 | 0.914 | 0.914 |
| right fronto-cingulate left motor-occipital-parietal | 221.011 | 235.550 | 0.349 | 0.781 |
| left fronto-cingulate right temporal | 27.539 | 42.800 | 0.521 | 0.781 |
| right fronto-cingulate left temporal | -9.478 | 21.281 | 0.657 | 0.844 |
| left temporal right motor-occipital-parietal | -91.957 | 108.829 | 0.399 | 0.781 |
| right temporal left motor-occipital-parietal | -286.764 | 140.863 | 0.043 | 0.193 |
| **Intra-hemispheric connection** | $\boldsymbol{\delta}$ | **SE** | **p-value** | **q-value** |
| left fronto-cingulate left temporal | -1138.861 | 586.779 | 0.054 | 0.225 |
| left fronto-cingulate left motor-occipital-parietal | -343.698 | 1123.016 | 0.760 | 0.760 |
| left temporal left motor-occipital-parietal | -2756.217 | 1581.689 | 0.083 | 0.225 |
| right fronto-cingulate right temporal | -974.896 | 611.831 | 0.113 | 0.225 |
| right fronto-cingulate right motor-occipital-parietal | -686.440 | 1115.276 | 0.539 | 0.647 |
| right temporal right motor-occipital-parietal | -1664.563 | 1803.591 | 0.357 | 0.536 |
| **Intra-modular connection** | $\boldsymbol{\delta}$ | **SE** | **p-value** | **q-value** |
| left fronto-cingulate | -6636.924 | 3203.536 | 0.039 | 0.207 |
| right fronto-cingulate | -3984.763 | 3729.899 | 0.286 | 0.430 |
| left temporal | 6582.062 | 5131.308 | 0.201 | 0.402 |
| right temporal | 548.183 | 5749.975 | 0.924 | 0.964 |
| left motor-occipital-motor | -253.806 | 5633.787 | 0.964 | 0.964 |
| right motor-occipital-motor | 10993.406 | 6017.274 | 0.069 | 0.207 |

**Supplemental Table 9. Longitudinal global cognitive composite effects in preHD: Cortico-striatal connections.** $\delta$ – parameter estimate of baseline cognitive composite effect, SE – standard error, p-value – probability valve, q-value – FDR corrected p-value. Longitudinal change was defined as a significantly superior fit for the full LMER compared to the reduced LMER omitting global cognitive composite * time interaction.

| Cortico-striatal connection (Connectome) | $\delta$ | SE | P-value | q-value |
|---|---|---|---|---|
| Left striatum fronto-cingulate | -431.959 | 444.913 | 0.333 | 0.909 |
| Right striatum fronto-cingulate | -218.826 | 378.821 | 0.564 | 0.909 |
| Left striatum temporal | -103.621 | 102.919 | 0.315 | 0.909 |
| Right striatum temporal | 47.646 | 180.734 | 0.792 | 0.909 |
| Left striatum motor-occipital-parietal | 18.488 | 158.160 | 0.907 | 0.909 |
| Right striatum motor-occipital-parietal | -16.283 | 142.786 | 0.909 | 0.909 |
| **Cortico-striatal connection (VCP)** | **$\delta$** | **SE** | **P-value** | **q-value** |
| Left striatum fronto-cingulate | 0.007 | 0.013 | 0.573 | 0.608 |
| Right striatum fronto-cingulate | 0.008 | 0.008 | 0.280 | 0.608 |
| Left striatum temporal | -0.004 | 0.008 | 0.574 | 0.608 |
| Right striatum temporal | 0.003 | 0.006 | 0.608 | 0.608 |
| Left striatum motor-occipital-parietal | -0.008 | 0.016 | 0.594 | 0.608 |
| Right striatum motor-occipital-parietal | 0.012 | 0.007 | 0.112 | 0.608 |

Track-On HD Investigators

A Coleman, J Decolongon, M Fan, T. Petkau (University of British Columbia, Vancouver); C Jauffret, D Justo, S Lehericy, K Nigaud, R Valabrègue (ICM and APHP, Pitié- Salpêtrière University Hospital, Paris). A Schoonderbeek, E P 't Hart (Leiden University Medical Centre, Leiden); DJ Hensman Moss, H Crawford, E Johnson, M Papoutsi, C Berna, D Mahaleskshmi (University College London, London). R Reilmann N Weber (George Huntington Institute, Munster); I Labuschagne (Monash University, Melbourne);B Landwehrmeyer, M Orth, I Mayer (University of Ulm, Ulm); H Johnson (University of Iowa).
